# Does a Change in Health Research Funding Policy Related to the Integration of Sex and Gender Have an Impact?

**DOI:** 10.1371/journal.pone.0099900

**Published:** 2014-06-25

**Authors:** Joy Johnson, Zena Sharman, Bilkis Vissandjée, Donna E. Stewart

**Affiliations:** 1 CIHR Institute of Gender and Health, University of British Columbia, Vancouver, British Columbia, Canada; 2 Faculty of Nursing, Université de Montréal, Montreal, Quebec, Canada; 3 University of Toronto and University Health Network, Toronto, Ontario, Canada; Tilburg University, Netherlands

## Abstract

We analyzed the impact of a requirement introduced in December 2010 that all applicants to the Canadian Institutes of Health Research indicate whether their research designs accounted for sex or gender. We aimed to inform research policy by understanding the extent to which applicants across health research disciplines accounted for sex and gender. We conducted a descriptive statistical analysis to identify trends in application data from three research funding competitions (December 2010, June 2011, and December 2011) (N = 1459). We also conducted a qualitative thematic analysis of applicants' responses. Here we show that the proportion of applicants responding affirmatively to the questions on sex and gender increased over time (48% in December 2011, compared to 26% in December 2010). Biomedical researchers were least likely to report accounting for sex and gender. Analysis by discipline-specific peer review panel showed variation in the likelihood that a given panel will fund grants with a stated focus on sex or gender. These findings suggest that mandatory questions are one way of encouraging the uptake of sex and gender in health research, yet there remain persistent disparities across disciplines. These disparities represent opportunities for policy intervention by health research funders.

## Introduction

It is increasingly recognised that scientific evidence often fails to account for sex and gender; consequently it is not always clear whether results can be equally applied to men and women. To address this gap funding agencies and journals are beginning to develop policies and approaches to enhance the uptake of sex and gender considerations by health researchers. The European Commission recently funded GENDER-NET, an ERA-NET project that involves nine national funding agency partners from across Europe who are considering ways to enhance the uptake of sex and gender considerations in research. Similarly, in 2012 the European Association of Science Editors struck a gender policy committee with a mandate to advance gender- and sex-sensitive reporting and communication in science, including the development of a common standard for journals [1].

In North America, the National Institutes of Health (NIH) has long had a policy on the inclusion of women and minority groups in clinical research, and in October 2014 will require applicants to “report their plans for the balance of male and female cells and animals in preclinical studies…with parallel changes in review activities and requirements” [2]. Internationally, numerous research funding agencies acknowledge the importance of sex and gender in their funding programmes; some, like the Irish Research Council, require applicants to explicitly describe the gender and sex dimensions of their proposed research [3]. Organizations like the US Institute of Medicine have hosted meetings to consider how sex and gender can be meaningfully integrated into science and editorial policy [4], [5]. In Canada, we have witnessed similar calls to change editorial policy [6]. The national health research funding agency, the Canadian Institutes of Health Research (CIHR), is a signatory on a 2009 federal health portfolio policy related to the necessity of accounting for gender and sex in policy and research and has been leading the way in implementing new practices in Canada.

In this paper, we analyze the impact of introducing a requirement that all applicants to CIHR indicate whether and how they are taking sex and gender into account in their research. Our intent is to highlight the extent to which applicants are considering sex and gender, identify areas of health research where sex and gender are well and poorly integrated, and reflect on opportunities to inform policy and practice aimed at fostering the inclusion of sex and gender in health research.

### Background and Context

CIHR is a signatory on the Government of Canada's Health Portfolio – Sex and Gender-Based Analysis Policy. This policy underscores the importance of integrating gender and sex into health research when appropriate, for there is significant evidence to “demonstrate that biological, economic and social differences between women and men contribute to differences in health risks, health services use, health system interaction and health outcomes” [7]. The CIHR Institute of Gender and Health defines sex and gender as follows:

*Sex* refers to a set of biological attributes in humans and animals. It is primarily associated with physical and physiological features including sex chromosomes, gene expression, hormone levels and function, and reproductive/sexual anatomy. Sex is usually categorized as female or male but there is variation in the biological attributes that comprise sex and how those attributes are expressed.

*Gender* refers to the socially constructed roles, behaviours, expressions and identities of girls, women, boys, men, and gender diverse people. It influences how people perceive themselves and each other, how they act and interact, and the distribution of power and resources in society. Gender is usually conceptualized as a binary (girl/woman and boy/man) yet there is considerable diversity in how individuals and groups understand, experience and express it.

Sex and gender influence the health of men, women, boys, girls, and gender diverse people, as evidenced by a large and growing body of literature establishing how the biological and the social intersect at every level, from the cellular to the societal [8], [9]. For example, an individual's bone density is shaped both by their biological makeup (e.g., hormone levels, genes) and by gendered social factors (e.g., clothing, occupation, physical activity level) [10], [11].

In recognition of the important influences of sex and gender on health, in December 2010 CIHR made a change to its grant application forms, requiring that all applicants respond to two questions: Are sex (biological) considerations taken into account in this study? Are gender (socio-cultural) considerations taken into account in this study? Initially only those responding in the affirmative to either question were asked to describe how sex and gender considerations would be taken into account in the proposed research design. This was changed after one funding cycle so that negative responders also had to provide an explanation. Descriptions are limited to 2,000 characters. (For the exact wording of the questions, see Table 1.) Applicants have access to a short web-based research guide on sex and gender [12] and a frequently asked questions document embedded in the online application system. These documents offer definitions of sex and gender and encourage applicants to define and operationalize these terms as appropriate to their research designs [13].

**Table 1:** Mandatory sex and gender questions.

1	Are sex (biological) considerations taken into account in this study? Yes/No
2	Are gender (socio-cultural) considerations taken into account in this study? Yes/No
3	If YES please describe how sex and/or gender considerations will be considered in your research design. (2000 character limit)
4	If NO please explain why sex and/or gender considerations are not applicable in your research design. (2000 character limit)

## Literature Review

The literature on the integration of gender and sex in health research spans research funding and policy [14], [15], research practice [16], [17] and research reporting [18], [19]. There is a related literature on the inclusion of women in science [20]–[23]. For the purposes of this paper, we are primarily interested in literature on research funding and policy. There is variation in the design and implementation of health research funding agency policies aimed at fostering the integration of sex and gender in research. Here we highlight two examples from the literature – the Netherlands Organization for Health Research and Development (ZonMw), which utilizes a diversity-focused approach, and the US National Institutes of Health, which utilizes an inclusion-focused approach.

In 1999, ZonMw implemented a policy stipulating that in order to receive financial support studies must address diversity factors, such as sex, age, and ethnicity in health research. Funding applicants and reviewers are provided with guidelines that contain general information about ZonMw's commitment to address diversity factors; in addition, instructions for grant applicants and reviewers highlight the inclusion of specific questions on diversity issues (including sex). A 2007 study showed that applicants who received program specific instructions on diversity were more likely to account for sex and other aspects of diversity (e.g., age, ethnicity) than those who received general guidelines [14].

In the United States, the NIH's policy is supported by legislation. Since 1993, the NIH Revitalization Act has required inclusion of women and minorities in clinical research as a condition of funding. The NIH *Guidelines on Inclusion of Women and Minorities as Subjects in Clinical Research* require principal investigators (PIs) to propose how they plan for the inclusion of women, minorities, and children in their research and, where relevant, to justify their exclusion. The guidelines apply to all biomedical and behavioural research sponsored by the NIH, especially Phase III clinical trials. It is the shared responsibility of ethics boards, scientific review groups and NIH program staff to evaluate whether the PI has adequately addressed these guidelines. Ethics boards must ensure NIH sponsored investigators provide adequate details on the composition of their study populations and appropriate justification for the exclusion of any particular sub-population. The study review group has the authority to penalize research proposals that fail to address adequate inclusion [24]. This requirement has been shown to increase reporting of sex-specific results in peer reviewed literature [18] [25], increase attention to the inclusion of women in clinical research among NIH Study Review Group members [23] [26], and increase women's participation as research subjects in NIH trials [27]. It has also been criticized for over-emphasizing inclusion and difference without getting at the underlying causes of health disparities [28]. The NIH is in the process of implementing additional requirements for sex-based inclusion in preclinical research designs [2].

CIHR's approach is policy-driven, in that the mandatory questions for applicants were implemented in response to a wider federal government policy on sex- and gender-based analysis. Similar to the approach taken by ZonMw, CIHR requires its applicants to answer mandatory questions on sex and gender; unlike the NIH, it does not mandate the inclusion of specific populations in research designs, nor does it have corresponding peer review criteria on sex and gender. In light of this hybrid approach, we set out to determine how researchers respond to a policy change that requires them to consider the relevance of sex and gender by responding to two questions when applying for research funding. In particular, we address the following questions:

1) In an open funding competition, how many researchers indicate they are considering sex and gender?

2) What areas of health science show evidence that sex and gender are well integrated or poorly integrated?

3) Are there particular themes in researchers' responses that could inform future policy and practice?

## Methods

The Open Operating Grant Program (OOGP) is the largest of CIHR's open calls for proposals, and provides operating funds to support all areas of health research. CIHR categorizes health research in four broad themes: (1) Biomedical, (2) Clinical, (3) Health Systems and Services, and (4) Social, Cultural and Environmental Factors that Affect the Health of Populations (truncated here as Population Health Research). We conducted a descriptive analysis of the OOGP applicants' responses to mandatory questions on the integration of sex and gender.

Proposals to the OOGP must align with CIHR's mission “to create new scientific knowledge and to enable its translation into improved health, more effective health services and products, and a strengthened Canadian health care system” [29]. The OOGP competitions are held twice a year, and in addition to a research proposal, applicants provide basic administrative data (including whether the nominated principal applicant identifies as female or male) and designate up to two of the peer review panels whose mandates they believe most closely align with their proposed research. Success rates for the OOGP currently hover around 17%.

We were interested in trends over time and conducted a qualitative analysis of a subset of responses. Included in the analysis were data gathered from funded applications from three competition cycles (December 2010, June 2011, and December 2011). We looked at trends across CIHR's four research themes as well as across CIHR's 50+ peer review panels organized by disciplines/topics. Panel members – who are researchers – review and numerically score OOGP applications.

## Results

We analysed data from three OOGP funding competitions: December 2010, June 2011, and December 2011. The total number of applications submitted for each competition was fairly consistent (N = 2,298, 2,294, and 2,285, respectively). In keeping with CIHR's privacy policies we analysed data only from applications that were successfully funded.

**Figure 1:**
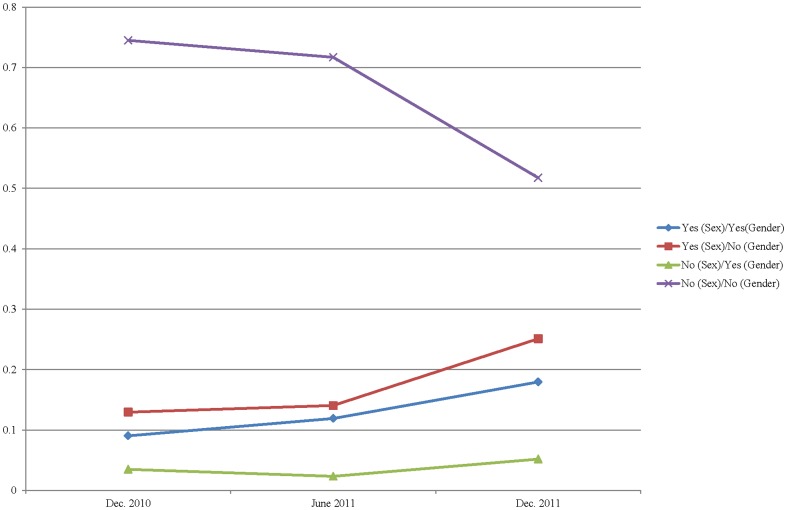
Percentage of responses to sex and gender items over three competitions. The proportion of respondents indicating that they did not consider sex or gender declined over time, while the proportion of respondents indicating that they considered sex and/or gender showed a corresponding increase. The addition of a requirement that respondents answering “no” provide a rationale for doing so appeared to correlate with an increase in the number of respondents answering in the affirmative.

There was an overall increase in the percentage of researchers responding affirmatively to the sex and gender questions over the course of the three competitions, which funded a total of 1459 projects (Fig. 1). The majority of funded projects were biomedical research (n = 955). The remainder were classified as clinical (n = 252), health systems (n = 102), or population health research (n = 150).

**Figure 2:**
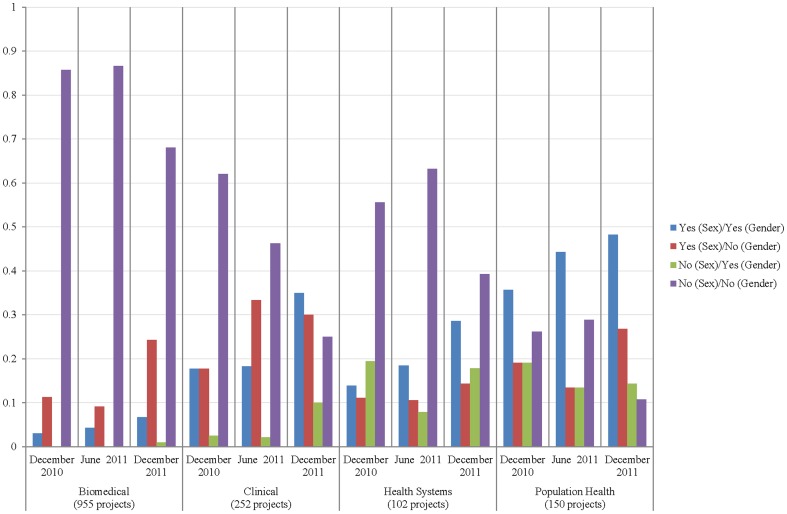
Applicant by research area, competition and responses to sex and gender items. We observed variation across disciplines, with applicants in the biomedical sciences being most likely to indicate that they were not integrating sex or gender in their research designs. Clinical researchers were most likely to account for sex, while population health researchers were most likely to account for gender.

The highest proportion of researchers indicating that they took sex into account was in the clinical research field, and the highest proportion indicating they were taking gender into account was in the population health field. Those in the biomedical field were more likely than others to indicate they took neither sex nor gender into account in their research with over 80% of respondents indicating this in December 2010 and June 2011 and over 60% in December 2011 (Fig. 2).

Overall, women PIs were more likely than men to indicate that their projects involved either sex (39% of women, 26% of men) or gender (25% of women, 12% of men) (Table 2). However, the percentage of men and women PIs who indicated both sex and gender was approximately equal in biomedical, clinical, and health system research areas, and more women (55%) than men (30%) in population health indicated both sex and gender (Table 2).

**Table 2:** Research area, by particpant sex and responses to sex and gender items.

Research Area	P.I. Sex	Y-Y		Y-N		N-Y		N-N		TOTAL	
**Biomedical**	**M**	38	5%	95	13%	2	0%	602	82%	**737**	**50%**
	**F**	6	3%	43	20%	1	0%	167	77%	**217**	**15%**
**Clinical**	**M**	33	22%	42	28%	5	3%	69	46%	**149**	**10%**
	**F**	25	25%	27	26%	7	7%	43	42%	**102**	**7%**
**Health Systems**	**M**	8	20%	5	13%	3	8%	24	60%	**40**	**3%**
	**F**	12	19%	7	11%	12	19%	31	50%	**62**	**4%**
**Population Health**	**M**	20	30%	16	24%	15	22%	16	24%	**67**	**5%**
	**F**	45	55%	14	17%	7	9%	16	20%	**82**	**6%**
**TOTAL**		**187**	**13%**	**249**	**17%**	**52**	**4%**	**968**	**66%**	**1456** *	**100%**

*3 records did not have the sex of primary investigator indicated.

Y-Y = “yes” to sex and “yes” to gender; Y-N = “yes” to sex “no” to gender; N-N = “no” to sex, “no” to gender.

We conducted a two-tailed binomial significance test to determine which set of applications to the 51 panels (Tables 3–5) diverged significantly from the overall rate at which proposals included a stated focus on sex or gender (specifically the 30% likelihood that an awarded grant would have a stated focus upon sex, and the 17% likelihood that an awarded grant would have a stated focus upon gender). Using an alpha level of α = 0.05, we found that 32 of the 51 panels diverged in at least one of the two focus areas. Seven panels were significantly more likely to award both grants focusing on sex and grants focusing on gender. These positive outlier panels included Aboriginal Peoples' Health; Biochemistry and Molecular Biology – B; Social and Developmental Aspects of Children's and Youth's Health; Gender, Sex and Health; Psychosocial, Sociocultural and Behavioural Determinants of Health (1 and 2); and Public, Community and Population Health (1). Three additional panels were identified which were significantly more likely to award grants pertaining to sex; five additional panels were significantly more likely to award grants pertaining to gender.

**Table 3:** CIHR panels exhibiting a significant tendency to award grants re: sex and/or gender.

Panel	Pertinent to Sex	Pertinent to Gender
Aboriginal Peoples' Health (ABH)	10 of 17	9 of 17
Biochemistry & Molecular Biology - B (BMB)	33 of 45	15 of 45
Social & Developmental Aspects of Children's & Youth's Health (CHI)	24 of 41	20 of 41
Gender, Sex & Health (GSH)	15 of 19	14 of 19
Psychosocial, Sociocultural & Behavioural Determinants of Health (PB1)	15 of 30	13 of 30
Psychosocial, Sociocultural & Behavioural Determinants of Health (PB2)	16 of 27	21 of 27
Public, Community & Population Health – (PH1)	18 of 31	13 of 31
Clinical Investigation - A (CIA) (Reproduction, Maternal, Children and Youth Health)	20 of 33	*6 of 33*
Nutrition, Food & Health (NUT)	20 of 37	*4 of 37*
Public, Community & Population Health – (PH2)	12 of 21	*7 of 21*
Humanities, Social Sciences, Law & Ethics in Health (HLE)	*8 of 20*	11 of 20
Health Policy & Systems Management Research (HPM)	*12 of 28*	13 of 28
Health Services Evaluation & Interventions Research (HS1)	*13 of 28*	12 of 28
Health Services Evaluation & Interventions Research (HS2)	*14 of 30*	13 of 30
Social Dimensions in Aging (SDA)	*7 of 13*	5 of 13

(Italicized items have a P-Value above 0.05/no significant tendency).

**Table 4:** CIHR panels exhibiting a significant tendency to not award grants re: sex and/or gender.

Panel	Pertinent to Sex	Pertinent to Gender
Biochemistry & Molecular Biology - A (BMA)	0 of 26	0 of 26
Cell Biology & Mechanisms of Disease (CBM)	3 of 34	0 of 34
Cell Physiology (CP)	0 of 27	0 of 27
Cancer Progression & Therapeutics (CPT a.k.a. CT2)	6 of 56	3 of 56
Developmental Biology (DEV)	1 of 22	0 of 22
Immunology & Transplantation (IT)	4 of 37	0 of 37
Systems & Clinical Neurosciences (NSA)	5 of 39	1 of 39
Molecular & Cellular Neurosciences (NSB)	3 of 31	1 of 31
Behavioural Sciences - B (BSB) (Clinical Behavioural Sciences)	1 of 18	*1 of 18*
Cancer Biology & Therapeutics (CBT)	2 of 30	*2 of 30*
Molecular & Cellular Biology of Cancer (MCC)	3 of 29	*1 of 29*
Microbiology & Infectious Diseases (MID)	3 of 30	*3 of 30*
Behavioural Sciences - A (BSA) (Behavioural Studies in Animal Models)	*6 of 30*	0 of 22
Clinical Investigation - B (CIB) (Arthritis, Bone, Skin and Cartilage)	*12 of 49*	2 of 49
Cardiovascular System - A (CSA) (Cells & Tissues)	*4 of 21*	0 of 21
Cardiovascular System - B (CSB) (Basic translational research in Cardiovascular Sciences)	*8 of 27*	0 of 27
Pharmaceutical Sciences (PS)	*6 of 38*	0 of 38

(Italicized items have a P-Value above 0.05/no significant tendency).

**Table 5:** CIHR panels not exhibiting a significant tendency re: sex and/or gender.

Panel	Pertinent to Sex	Pertinent to Gender
Biological & Clinical Aspects of Aging (BCA)	*11 of 35*	*4 of 35*
Biomedical Engineering (BME)	*3 of 26*	*1 of 26*
Behavioural Sciences - C (BSC) (Behavioural Studies and Neural Imaging)	*9 of 28*	*5 of 28*
Clinical Investigation – C (CIC)	*9 of 19*	*2 of 19*
Clinical Investigation - D (CID) (Translational Research in Cardiovascular Sciences)	*11 of 25*	*5 of 25*
Diabetes, Obesity, Lipid & Lipoprotein Disorders (DOL)	*11 of 40*	*4 of 40*
Endocrinology (E)	*9 of 18*	*0 of 18*
Genetics (G)	*14 of 38*	*4 of 38*
Genomics (GMX)	*1 of 15*	*0 of 15*
Haematology, Digestive Disease & Kidney (HDK)	*4 of 29*	*2 of 29*
Knowledge Translation Research (KTR)	*1 of 16*	*1 of 16*
Movement & Exercise (MOV)	*6 of 29*	*1 of 29*
Medical Physics & Imaging (MPI)	*3 of 26*	*2 of 26*
Palliative & End of Life Care (PLC)	*2 of 9*	*3 of 9*
Pharmacology & Toxicology (PT)	*7 of 21*	*3 of 21*
Randomized Controlled Trials (RC1)	*9 of 22*	*4 of 22*
Respiratory System (RS)	*8 of 30*	*2 of 30*
Virology & Viral Pathogenesis (VVP)	*14 of 55*	*7 of 55*

(All tendencies have a P-Value above 0.05/no significant tendency).

Eight panels were significantly less likely to award grants focusing on either sex and gender. These were: Biochemistry & Molecular Biology – A; Cell Biology and Mechanisms of Disease; Cell Physiology; Cancer Progression and Therapeutics; Developmental Biology; Immunology and Transplantation; Systems and Clinical Neurosciences; and Molecular and Cellular Neurosciences. Four additional panels were determined to be significantly less likely to award grants focusing on sex, and five additional panels were determined to be significantly less likely to award grants focusing on gender. The 19 remaining panels did not significantly diverge from the normal distribution in either of the values measured. These results suggest that the integration of sex and gender is divided upon disciplinary lines, with the behavioural and public health communities having adopted the integration sex/gender and those panels based on cellular processes having apparently resisted voluntary incorporation of these considerations.

We conducted a content analysis of the responses to the questions and the accompanying abstract for 200 randomly selected funding records from the 1459 successful grants, and categorized them by pattern of response to the sex and gender questions (e.g., yy, yn, ny, nn). Of this sample, twenty-six applicants indicated that their design integrated both sex and gender, 34 indicated that their design integrated sex, seven indicated that their design integrated gender, and 133 indicated that their design did not integrate sex or gender. We were most interested in determining patterns in responses, whether responses were appropriate and justified, and if any areas of science seemed particularly recalcitrant to notions of sex and gender.

### Conflation of Sex and Gender

In most categories, there was evidence of applicants using the terms sex and gender interchangeably. For example, applicants used the term “gender” in reference to animal model-based studies focused on sex and in relation to investigations of biological differences: “An equal number of male and female animals will be studied to avoid any [genetic] bias resulting from gender differences.” Interestingly, we were more likely to see gender used as a synonym for sex, however, studies primarily focused on gender did not tend to conflate it with sex.

### If You Are Studying Women You Are Studying Sex/Gender

Among the proposals that integrated sex and/or gender, we observed respondents equating a sampling parameter with a sex- and/or gender-based analysis. We saw this pattern in studies with samples composed only of males or females, or in studies with sampling parameters that included both males and females (be they cell lines, animals, or humans). For example, a proposed study with a sample composed only of women or an equal number of men and women was given as an explanation of how sex/gender was integrated (e.g., “We will recruit an equal number of women and men…in this study”). However, in these rationales, the descriptions of the methods did not specify a plan for analyzing these data by sex and/or gender.

### Sex/Gender as a Covariate

A number of abstracts for studies that indicated there was an integration of sex referred to sex as a covariate, stratifying by sex or controlling for sex, or to strategies for avoiding differential item functioning on gender variables. For example, “At a population level, girls and women are consistently less physically active than boys and men. Accordingly, our individual level analyses will control for sex.” Several of the abstracts describing designs that incorporated the use of secondary data focused on sex, based on the conviction that these data are better able to capture sex than gender, while others were able to make a compelling case for using the categories of male and female as proxies for both sex and gender.

### Sex/Gender Are Not Relevant

Among the studies that did not integrate sex or gender, many respondents used the justification that sex or gender were not relevant to basic science or to non-human research focused on animal or cell models. As one respondent succinctly put it, “This is a basic science research project.” As another explained, “No human subjects used in this study.” Some respondents indicated that they were not considering sex or gender because the issue at hand was “equally important” or affected both men and women. Others indicated that sex and gender were not relevant because they had a single-sex sample (only males/only females), thereby suggesting that we can only study sex or gender by comparing females and males. For example, “Sex and/or gender are not applicable in this research proposal, the particular [condition] is only associated with men;” “This study is being performed entirely in women.” One respondent deemed sex and gender not relevant because their research focused on system-level factors. We also observed a number of respondents justifying the omission of sex and gender on the basis of a lack of evidence – for example, no prior evidence of sex or gender-based differences.

## Conclusions

Our analysis showed an overall increase in the proportion of CIHR-funded researchers incorporating sex and gender in their research designs. This trend varied by discipline, with biomedical researchers being least likely to account for sex and gender, clinical researchers being most likely to account for sex, and population health researchers being most likely to account for gender. Women PIs were more likely to respond in the affirmative than their male counterparts, adding a potentially intriguing dimension to ongoing efforts to foster the participation of women in science and how these efforts might intersect with efforts to foster the integration of sex and gender in health research [20], [22], [23].

Our study had several limitations. Our analysis was confined only to successful applicants because privacy requirements prevented us from analyzing data for unsuccessful applicants. Given that CIHR's funding success rate currently ranges between 15-19%, this excludes a considerable proportion of the applicant pool. Moreover, our analysis was limited to data from the three funding competitions after implementation of the mandatory questions on sex and gender (December 2010, June 2011, and December 2011). As such, the findings reported here are from a relatively short period. At a broader level, we realize the need for caution in making causal claims concerning the impact of changing guidelines. Increasing attention to gender and sex in health research is surely the result of a constellation of factors, including broader societal trends and shifting understandings of what constitutes scientific excellence.

Indeed, the trends we observed in our data reflect broader trends in health research and in science. Reviews of the literature continue to show failure to account for sex and gender in research in fields such as neuroscience and biomedicine [30], [11], public health [31] and other domains, despite considerable evidence for the scientific and clinical importance of integrating sex and gender in health research [32], [33]. These reviews share a common thread – that is, a call to action aimed at changing health research practices. Strategies to foster the inclusion of sex and gender in health research have had success, yet the literature reveals a clear knowledge gap: although women are increasingly being included in clinical trials, studies monitoring policies aimed at fostering the integration of sex and gender considerations have demonstrated that the inclusion of both sexes in a clinical trial does not automatically lead to the analysis and reporting of data by sex [17], [18]. Indeed, specific analysis and reporting of sex-specific results remains limited [34], although it is well documented that a lack of knowledge about sex differences results in knowledge gaps in health research [35], [36].

To address the lack of analysis and reporting of gender- and sex-specific results, there has been discussion of the role of journals in emphasizing the importance of accounting for sex and gender in health research, and a call for international journals to require authors to report results by sex and gender [37]. Heidari and colleagues [19] recommend uniform requirements for manuscripts submitted to biomedical journals, emphasizing the ethical obligation of authors to present data analyzed by gender and sex, and the urgency for journal editors to promote ethical and adequate standards of reporting by integrating requirements for inclusion of sex and gender analysis. Geller and colleagues [17] state the effort to ensure enhanced inclusion, analysis, and reporting of sex and gender must be made in partnership between health research agencies, journal editors, and researchers themselves.

As we have shown in this paper, funding agencies have a key role to play in enabling this shift [38]. For example, the design and implementation of funding agency-level changes such as extending sex-based inclusion requirements to preclinical animal studies, providing applicants with clear instructions on sex and gender, educating applicants, peer reviewers and agency staff on the importance of sex and gender, and engaging in regular measurement and monitoring of progress [15], [39]. At CIHR we are developing a suite of training materials on sex and gender for health researchers and peer reviewers. This builds on past initiatives such as our gender, sex and health research casebook [40]. It reflects our interest in attending to the knowledge gaps suggested by the results of the qualitative analysis presented here – for example, the persistent conflation of sex and gender by health researchers, the assumption that gender applies only to women, and the perception that sex is not relevant to research on animal or cell models.

As we develop and launch training materials and other initiatives we will continue to grapple with the challenge of how to enable meaningful and appropriate integration of sex and gender throughout the health research process. Our approach to date has included the implementation of the mandatory questions, researcher and peer reviewer education, and outreach to journals, among other activities and initiatives. It should be noted that the mandatory questions are not associated with mandatory peer review criteria – that is, peer reviewers are not required to score applicants on their answers to the sex and gender questions. We recognize that the implementation of mandatory questions without corresponding peer review criteria has its limitations, in that it may inspire researchers to reflect on how sex and gender figure into their research designs without necessarily resulting in tangible changes in research practice. It is for this reason that we are engaging in parallel efforts to train researchers and peer reviewers on the integration of sex and gender, as well as collaborating nationally and internationally on the development of sex and gender reporting requirements for journals. All of our forthcoming initiatives will build on what we have learned from our findings, in recognition of the importance of tailoring our tools and offerings to the appropriate scientific communities and their research practices.
